# Stronger cortisol response to acute psychosocial stress is correlated with larger decrease in temporal sensitivity

**DOI:** 10.7717/peerj.2061

**Published:** 2016-05-26

**Authors:** Zhuxi Yao, Liang Zhang, Caihong Jiang, Kan Zhang, Jianhui Wu

**Affiliations:** 1 Key Laboratory of Behavioral Science, Institute of Psychology, Chinese Academy of Sciences, Beijing, China; 2 University of Chinese Academy of Sciences, Beijing, China; 3 Institute of Human Factors & Ergonomics, Department of Industrial Engineering, Tsinghua University, Beijing, China

**Keywords:** Cortisol, Acute stress, Temporal bisection, Temporal sensitivity, The Trier Social Stress Test, Time perception

## Abstract

As a fundamental dimension of cognition and behavior, time perception has been found to be sensitive to stress. However, how one’s time perception changes with responses to stress is still unclear. The present study aimed to investigate the relationship between stress-induced cortisol response and time perception. A group of 40 healthy young male adults performed a temporal bisection task before and after the Trier Social Stress Test for a stress condition. A control group of 27 male participants completed the same time perception task without stress induction. In the temporal bisection task, participants were first presented with short (400 ms) and long (1,600 ms) visual signals serving as anchor durations and then required to judge whether the intermediate probe durations were more similar to the short or the long anchor. The bisection point and Weber ratio were calculated and indicated the subjective duration and the temporal sensitivity, respectively. Data showed that participants in the stress group had significantly increased salivary cortisol levels, heart rates, and negative affects compared with those in the control group. The results did not show significant group differences for the subjective duration or the temporal sensitivity. However, the results showed a significant positive correlation between stress-induced cortisol responses and decreases in temporal sensitivity indexed by increases in the Weber ratio. This correlation was not observed for the control group. Changes in subjective duration indexed by temporal bisection points were not correlated with cortisol reactivity in both the groups. In conclusion, the present study found that although no significant change was observed in time perception after an acute stressor on the group-level comparison (i.e., stress vs. nonstress group), individuals with stronger cortisol responses to stress showed a larger decrease in temporal sensitivity. This finding may provide insight into the understanding of the relationship between stress and temporal sensitivity.

## Introduction

Individuals differ dramatically in their psychophysiological responses to stress, and their brain functions and behavioral performances also vary with their stress responsiveness (Pruessner et al., 1997; Lupien et al., 2007; Sapolsky, 2015). For example, Starcke and colleagues (2011) found large interindividual differences in endocrine stress reactions and an association between individual’s cortisol response to stress and decision-making behavior, although no significant behavioral changes in decision-making were found under stress compared with the nonstress control condition. Time perception is a fundamental dimension of cognition and behavior (Buhusi & Meck, 2005). Studies have shown that time perception is crucial for many fundamental functions, e.g., motor control (Edwards, Alder & Rose, 2002) and acoustic communication signals, including human speech and music (Mauk & Buonomano, 2004; Schirmer, 2004). These functions rely on precise temporal perception and play important roles for everyday functioning (Buhusi & Meck, 2005). Maintaining accurate and sensitive time perception is essential for individuals’ adaptation to the changing environment, especially when facing stressful conditions (Hancock & Weaver, 2005; Eisen, 2009). However, how one’s time perception alters with responses to stress is still unknown.

Accuracy and precision are two elementary aspects of time perception. The subjective duration and the temporal sensitivity are the index of these two aspects, respectively. The former refers to one’s perceived duration of a certain stimulus interval, and the latter refers to one’s ability to discriminate between different durations (Meck, 1983; Grondin & Rammsayer, 2003; Kopec & Brody, 2010). Literature on time perception has suggested disassociated underlying mechanisms for subjective duration and temporal sensitivity (Grondin & Rammsayer, 2003; Cheng & Meck, 2007; Rammsayer, 2010; Stauffer et al., 2012; Yin & Meck, 2014). This assumption was supported by the dissociated performance on perceived durations and discrimination on a behavioral level (Rammsayer, 2010). On a neurological level, it has been found that lesions of the dorsal hippocampus decreased the subjective duration with the temporal sensitivity unaffected (Yin & Meck, 2014).

Researchers have proposed cognitive models and neural mechanisms for time perception. In cognitive models, time perception is assumed to be based on three processing stages: clock stage, memory stage, and decision stage (Church, 1984; Zakay & Block, 1997; Grondin, 2010; Kopec & Brody, 2010). Temporal information is first encoded in the clock stage which consisting of a pacemaker that emits pulses at a given rate, a switch controlling pulse transfer, and an accumulator in which the number of pulses is stored (Droit-Volet & Meck, 2007). Attention and arousal play important roles in the clock stage (Zakay & Block, 1997). Representations of the current passage of time are then stored into working memory and/or long-term memory, and compared with those stored in long-term memory to generate a judgment in the decision stage at last (Church, 1984; Grondin, 2010). The subjective duration may depend mainly on the pacemaker-accumulator process, on which arousal and attention would exert a critical influence (Grondin & Rammsayer, 2003). The temporal sensitivity would be more closely associated with higher order components of the discrimination process, and memory and decisional components may be involved in these processes (Grondin & Rammsayer, 2003; Wearden & Ferrara, 1993). The prefrontal–striatal–hippocampal circuits have been suggested to support time perception in the range of hundreds of milliseconds to multi-seconds (Buhusi & Meck, 2005; Coull, Cheng & Meck, 2011; Gu, Van Rijn & Meck, 2015). The prefrontal cortex is implicated in encoding of the current durations and comparison between the current and the memorized durations (Coull, Cheng & Meck, 2011; Gu, Van Rijn & Meck, 2015). The striatum is critical in the representation of temporal information (Buhusi & Meck, 2005; Meck, Church & Matell, 2013). The hippocampus cooperates with these brain regions to form the representation of temporal memory (Coull, Cheng & Meck, 2011; Meck, Church & Matell, 2013).

Cognitive components of time perception have been found to be sensitive to acute stress and to vary with cortisol responses to stress (Buchanan & Tranel, 2008; Buchanan, Tranel & Adolphs, 2006; Qin et al., 2009; Schoofs, Wolf & Smeets, 2009). Previous studies found that participants with greater stress-induced cortisol increases performed worse in working memory tasks (Qin et al., 2009; Schoofs, Wolf & Smeets, 2009). Higher cortisol responses to stress were associated with larger long-term memory impairments (Buchanan & Tranel, 2008; Smeets, 2011). Negative correlations between male individual’s cortisol stress responses and disadvantageous decisions were also reported. The male participants with higher cortisol responses to stress showed poorer performance in the subsequent decision-making task (Van den Bos, Harteveld & Stoop, 2009).

Meanwhile, brain regions involved in time perception (Coull, Cheng & Meck, 2011; Gu, Van Rijn & Meck, 2015), especially the prefrontal cortex and hippocampus, are main targets of the stress hormones including cortisol (McEwen, 2004; Lupien et al., 2007; Arnsten, 2009). More importantly, functional activities of theses brain regions also showed a correlation with stress-induced cortisol responses. For example, cortisol increases to stress were found to be related to reduced activation in dorsolateral prefrontal cortex during a working memory task (Qin et al., 2009). Studies also found a positive correlation between the degree of deactivation in the hippocampus and the release of cortisol in response to the stress task (Pruessner et al., 2008).

Previous studies investigating the relationship between stress and time perception have focused on comparing differences between stress condition and control condition. Subjective durations under acute stress were found to be longer than those under control condition (Meck, 1983; Watts & Sharrock, 1984; Campbell & Bryant, 2007; Droit-Volet et al., 2010; Tamm et al., 2014). However, mixed results were found regarding temporal sensitivity under acute stress. In an early study, Watts & Sharrock (1984) found that acute stress decreased temporal precision. However, two recent studies showed increased temporal sensitivity under stress condition relative to the control condition (Tamm et al., 2014; Tamm et al., 2015). In addition, there was also the study reporting that acute stress did not affect temporal sensitivity (Droit-Volet et al., 2010). One of the reasons of these complex results between groups might be the large individual difference in the effect of stress on time perception. As the previous studies showed, individual differences in responses to stress are enormous (Lupien et al., 2007), and the impairments of stress on the cognitive components, including memory and decision making, varies a lot among individuals (e.g., Qin et al., 2009; Smeets, 2011). According to the framework of the cognitive models, the temporal sensitivity is associated with memory and decisional components (Grondin & Rammsayer, 2003). Some individuals may have severe impairments on these components under stress, and thus show imprecise temporal perception. But these cognitive components of other individuals may not be affected by stress and these individuals can remain good temporal sensitivity. Therefore, besides the comparisons between the groups or conditions, investigations on the correlations between the individuals’ stress responses and time perception are needed. However, these relationships have not yet been investigated.

Therefore, the present study aimed to investigate time perception changes under psychosocial stress and, most importantly, the relationship between time perception and stress responses. For this purpose, the Trier Social Stress Test (TSST; Buchanan, Tranel & Kirschbaum, 2009; Kirschbaum, Pirke & Hellhammer, 1993) was used as an acute psychosocial stressor to elicit cortisol responses, and a control group was included. Time perception was assessed before and after treatment using a temporal bisection task (Droit-Volet et al., 2010; Droit-Volet, Fayolle & Gil, 2011), which is one of the most commonly used tasks to measure the subjective durations and temporal sensitivity (Allan & Gibbon, 1991; Wearden, 1991; for reviews, see Kopec & Brody 2010; Wearden & Jones, 2013). Based on the aforementioned literature review, it was expected that the time perception performance would change under the acute psychosocial stress. Specifically, we hypothesized that longer subjective duration and/or altered temporal sensitivity would be found in the TSST condition. We also predicted that the stronger cortisol responses to stress would be correlated to the decline in temporal sensitivity and/or extension in subjective duration.

## Methods

### Participants

Sixty-seven healthy male students (age range: 18–24 years; mean (*M*): 21.22 years; standard deviation (*SD*): 1.28) were recruited in the present study from different universities in Beijing by advertising online. Due to a potential influence on stress responses, the following exclusion criteria were employed: (1) cold or any medication use within 2 weeks of participation in the study; (2) chronic use of any psychiatric, neurological, or endocrine medication; (3) any major chronic physiological disease; (4) any history of psychiatric, neurological, or endocrine disorders; (5) chronic overnight work or circadian disruption; and (6) current periodontitis. Besides, individuals who had excessive alcohol consumption (more than two alcoholic drinks daily) or nicotine consumption (more than five cigarettes a day) were excluded from recruitment because ample evidence showed that alcohol and nicotine (for a review, see Kudielka, Hellhammer & Wust, 2009) influence stress responses. All participants had normal or corrected-to normal vision and were right handed.

The participants were randomly assigned to either the stress or the control condition. Five participants in the stress condition and three participants in the control condition had missing data of salivary cortisol due to insufficient salivary volume or outliers in data of one or more measurements (3 *SD*), and thus were excluded from the analysis. In addition, two stress nonresponders in the stress group, that is, whose cortisol changes from the baseline to the peak of cortisol response (25 min after the onset of TSST; Dickerson & Kemeny, 2004) were equal to or less than zero, were excluded (Buchanan, Tranel & Adolphs, 2006; Buchanan & Tranel, 2008; Merz, Wolf & Hennig, 2010; Rimmele & Lobmaier, 2012). This resulted in a total of 57 participants with 33 in the stress group and 24 in the control group. The two groups in this final sample did not differ significantly in age (stress group: *M* = 21.39 years, *SD* = 1.35; control group: *M* = 21.21 years, *SD* = 1.22; *p* = .595), body mass index (stress group: *M* = 21.89 kg/m^2^, *SD* = 1.86; control group: *M* = 21.48 kg/m^2^, *SD* = 2.05; *p* = .443), and years of education (stress group: *M* = 14.73 years, *SD* = 0.57; control group: *M* = 14.58 years, *SD* = 0.83; *p* = .442). This experiment was approved by the Ethics Committee of Human Experimentation in the Institute of Psychology, Chinese Academy of Sciences. All participants gave written informed consent and were paid for their participation.

### General procedure

**Figure 1:**
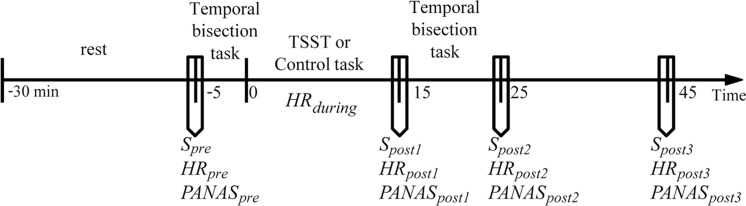
General procedure of the experiment. Timeline depicting the saliva sampling(S) procedure, the heart rate (HR) recording procedure, the affective state measurement procedure (the Positive and Negative Affect Schedule, PANAS), the pre-treatment temporal perception task, stress induction (the Terier Social Stress Test, TSST) or control task, and post-treatment temporal perception task.

Before coming to the laboratory, all participants were required not to drink or eat anything besides water and not to do vigorous exercise for 2 h. All participants reported to have complied with these requirements. To control for the circadian rhythm of cortisol levels (Dickerson & Kemeny, 2004; Kudielka et al., 2004), all participants were tested between 2:00 pm and 5:00 pm. Upon arrival, the participants were introduced briefly about the experiment. They were then required to complete a questionnaire of basic information and allowed to relax in a quiet room for 25 min. The heart rate (*HR*_*pre*_), the salivary sample (*S*_*pre*_), and the positive and negative affective states (*PA*_*pre*_ and *NA*_*pre*_) were measured. The participants completed the first session of the temporal bisection task (pretreatment session). The participants were randomly assigned to either the stress or the control condition. During the stress induction or control task (15 min in total), the heart rate was continuously recorded (*HR*_*during*_). Immediately after the treatment, the heart rate (*HR*_*post*1_), the salivary sample (*S*_*post*1_), and the positive and negative affect states (*PA*_*post*1_ and *NA*_*post*1_) were measured again. Then, the participants completed the second session of the temporal bisection task (post-treatment session, which was the same as the pretreatment session). The heart rate, the salivary sample, and the positive and negative affect states were measured again at +25 min and +45 min relative to the onset of the stress/control condition. The general procedure of the experiment is depicted in Fig. 1.

### Stress induction and control condition

#### Stress induction

Acute psychosocial stress was induced by a modified version of TSST (Kirschbaum, Pirke & Hellhammer, 1993; Buchanan, Tranel & Kirschbaum, 2009; Buchanan et al., 2012). The modified TSST was as effective as or even more effective in eliciting cortisol responses than the original TSST (Buchanan, Tranel & Kirschbaum, 2009; Buchanan et al., 2012), consisting of a preparation period (5 min) and a test period (10 min) during which the participants delivered a speech (5 min) and performed mental arithmetic (5 min) in front of an “audience” of experimenters. In the scenario of the speech, the participants were instructed to imagine that they were accused of shoplifting and had to defend themselves in front of the store managers. During the mental arithmetic task, the participants were required to serially subtract the number 13 from 1,022 as fast and as accurately as possible. They had to restart at 1,022 once they made an error. After the preparation in laboratory room A, the participants were escorted to laboratory room B to complete the speech and mental arithmetic tasks. Three experimenters (two females and one male) were present throughout the Test period. They wore white coats, kept a neutral expression, withheld facial and verbal feedback, and communicated with the participants in a neutral manner. The participants stood in front of the experimenters and spoke into a microphone and a video camera throughout.

#### Control condition

The control task contained similar tasks (verbal and numeric) designed to be analogous to the TSST in time course and cognitive load, but not stressful with the social and self-relevant components omitted (Het et al., 2009). The procedures in the control condition were similar to the original version (see Het et al., 2009) but modified in aspect of the speech content (based on Buchanan, Laures-Gore & Duff, 2014). As in the TSST group, the control group had a 5-min period for preparation in laboratory room A, during which the participants were instructed to read a general interest travel article and prepare a summary of its contents. During the speech task, the participants remained seated in laboratory room A to deliver a 5-min speech of the prepared summary into a video camera without any experimenter present. The participants were then asked to complete a simple written arithmetic task for 5 min.

### Temporal bisection procedure

The settings and procedure of the temporal bisection task were as described in previous studies (Droit-Volet et al., 2010; Droit-Volet, Fayolle & Gil, 2011; Yao et al., 2015). Each participant completed two sessions of the temporal bisection task with exactly the same procedure. The first session was given before the treatment (pretreatment), and the second was given 5 min after the end of the treatment (post-treatment). For each session, the participants were first presented with the short (400 ms) and long anchor duration (1,600 ms) once in a random order. Then, they completed eight blocks of seven trials (a total of 56 trials), with each probe duration (400, 600, 800, 1,000, 1,200, 1,400, or 1,600 ms) presented once in a random order within each block. For each trial, the word “ready” was first presented for 500 ms, immediately followed by a blank screen of 200 ms, and then the blue circle (presenting the probe duration, 2.5 cm in diameter) was presented in the center of the screen. The participants were required to judge whether the probe duration was more similar to the short or long anchor duration by pressing the corresponding key with the index fingers of their right or left hand. The inter-trial interval was random from 500 to 1,500 ms. All participants were instructed not to use any strategy to count time, such as counting numbers or tapping (Clement & Droit-Volet, 2006). The procedure was run using E-prime (Psychological Software Tools Inc., PA, USA).

### Physiological measures

Saliva sample was collected using Salivette collection tubes (Sarstedt, Rommelsdorf, Germany). Within 40 min after collection, the saliva sample was frozen at −22 °C until analysis. Sample was thawed and centrifuged at 3,000 rpm for 10 min. Cortisol concentration was measured using electrochemiluminescence immunoassay (Cobas e 601, Roche Diagnostics, Numbrecht, Germany). The lower sensitivity for cortisol was 0.5 nmol/L. Intra- and inter-assay variations were below 10%.

Heart rate (HR) was measured using the electrocardiogram module of Biopac Amplifier-System (MP150; Biopac, Goleta, CA, USA) with three electrocardiograph electrodes placed on the skin, one placed on the right side of the neck and the other two on the left and right inner ankles, respectively. Signals were recorded at a sample rate of 1,000 Hz. The pre- and post-treatment measures of the HR (*HR*_*pre*_, *HR*_*post*1_, *HR*_*post*2_, and *HR*_*post*3_) were recorded continuously for 5 min each, and the measure of HR during treatment (*HR*_*during*_) was recorded continuously for 15 min throughout the stress induction or control task. The HR was averaged across each measuring period using the AcqKnowledge software and defined as the number of beats per minutes (bpm).

### Psychological measures

Positive and negative affective states were assessed using the Positive and Negative Affect Schedule (PANAS; Watson, Clark & Tellegen, 1988) consisting of 10 items for positive affects (PA, e.g., “interested,” “enthusiastic”) and 10 items for negative affects (NA, e.g., “upset,” “ashamed”) describing current affect. Answers were given on a 5-point scale from 1 “very slightly or not at all” to 5 “extremely.” The ratings were summed up to a score for PA and a score for NA both ranging from 10 (minimum) to 50 (maximum).

### Data management and analysis

To examine whether the stress induction procedure was effective, mixed two-way analysis of variances (ANOVAs) were conducted for salivary cortisol, HR, and affective states by including Group (stress, control) as between-subjects variable and Test period as within-subjects variable. Note that the number of levels for the Test period differed across dependent variables, five levels for HR, and four levels for salivary cortisol, PA, and NA.

For time perception performance, *P*(long), bisection point (BP), and Weber ratio (WR) were calculated. *P*(long) is the proportion of “long” responses for each probe duration. For example, if participants judged 35% of the 800-ms probes to be long, the *P*(long) for the 800-ms probe would be 35%. To calculate the BP and WR, the logistic function, *P*(long) = 1/[1 + exp(a*Duration +*b*)], was first fitted to individual participant data, in which Duration stood for probe duration, and *a* and *b* were free parameters (Droit-Volet et al., 2010). The BP was defined as the probe duration that was judged as long with a 50% probability (Duration [*P*(long) = 50%]), which indicated subjective duration. The lower the BP is, the longer the subjective duration. To calculate the WR, the probe durations that were judged as long with 25% and 75% probability, that is, (Duration [*P*(long) = 25%]) and (Duration [*P*(long) = 75%]), were first obtained. Then, the WR was calculated using the following expression: WR = (Duration [*P*(long) = 75%] − Duration [*P*(long) = 25%])/(2*BP) (Meck, 1983). The WR indicates temporal sensitivity, with a lower WR value indicating a higher temporal sensitivity.

A mixed three-way ANOVA with Probe duration (400, 600, 800, 1,000, 1,200, 1,400, and 1,600 ms) and Test period (pretreatment, post-treatment) as within-subjects variables and Group (stress, control) as between-subjects variable was conducted for *P*(long). Also, two separate mixed two-way ANOVAs for the BP and the WR were conducted with Test period (pretreatment, post-treatment) as within-subject variable and Group (stress, control) as between-subjects variable.

To investigate whether stress-induced cortisol responses were correlated with temporal performance changes, Pearson correlation was conducted between salivary cortisol responses and temporal performance changes in the stress group, including changes in BPs (BP_*post*_ minus BP_*pre*_) and changes in WRs (WR_*post*_ minus WR_*pre*_). Individual salivary cortisol responses were calculated by the area under the curve with respect to the increase (AUCi) in salivary cortisol concentration: ${\mathrm{AUC}}_{\mathrm{i}}= \frac{1}{2} \cdot \left( {S}_{pre}+{S}_{post1} \right) \cdot {t}_{{S}_{pre}-{S}_{post1}}+ \frac{1}{2} \cdot \left( {S}_{post1}+{S}_{post2} \right) \cdot {t}_{{S}_{post1}-{S}_{post2}}+ \frac{1}{2} \cdot \left( {S}_{post2}+{S}_{post3} \right) \cdot {t}_{{S}_{post2}-{S}_{post3}}-{S}_{pre}\cdot \left( {t}_{{S}_{pre}-{S}_{post1}}+{t}_{{S}_{post1}-{S}_{post2}}+{t}_{{S}_{post2}-{S}_{post3}} \right) = \frac{1}{2} \cdot \left( {S}_{pre}+{S}_{post1} \right) \cdot \left( \frac{1}{3} \right) + \frac{1}{2} \cdot \left( {S}_{post1}+{S}_{post2} \right) \cdot \left( \frac{1}{6} \right) + \frac{1}{2} \cdot \left( {S}_{post2}+{S}_{post3} \right) \cdot \left( \frac{1}{3} \right) -{S}_{pre}\cdot \left( \frac{1}{3} + \frac{1}{6} + \frac{1}{3} \right) $, in which *t* denotes the time interval between two successive salivary samplings expressed in hour (Pruessner et al., 2003). Also, the same analyses were performed for the control group.

Greenhouse–Geisser correction was used when the requirement of sphericity in the ANOVA for repeated measures was violated. The *η*^2^ measure of effect size was included where appropriate. Post hoc comparisons were conducted using the Bonferroni adjustments for the *p* values. All reported *p* values were two-tailed, and the level of significance was set at .05. The statistical analyses were performed using SPSS 18.0.

## Results

### Stress responses

**Figure 2:**
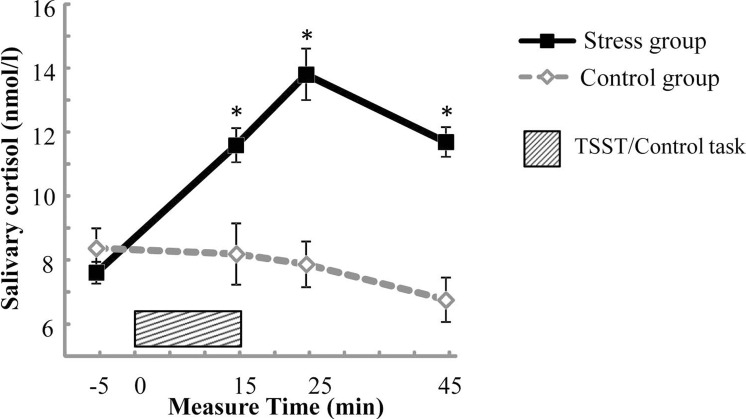
Salivary cortisol concentrations across the experiment for the stress group and the control group. Error bars are SEM. * denotes significant group difference (*p* < 0.05).

For salivary cortisol, the mixed two-way ANOVA revealed significant main effects of Test period, *F*(3, 165) = 14.742, *p* < .001, partial *η*^2^ = .211, and Group, *F*(1, 55) = 20.272, *p* < .001, partial *η*^2^ = 0.269, and a significant interaction of Test period * Group, *F*(3, 165) = 22.425, *p* < .001, partial *η*^2^ = 0.290. Simple effects analysis revealed that the baseline salivary cortisol level (*S*_*pre*_) in the stress group was not significantly different from that in the control group (*p* > .05), and salivary cortisol levels (*S*_*post*1_, *S*_*post*2_, and *S*_*post*3_) measured after treatment were significantly higher in the stress group compared with those in the control group (*ps* < .01) (see Fig. 2).

For HR, the mixed two-way ANOVA revealed a significant main effect of Test period, *F*(4, 220) = 141.644, *p* < .001, partial *η*^2^ = 0.720, and a significant interaction of Test period * Group, *F*(4, 220) = 43.338, *p* < .001, partial *η*^2^ = 0.441. Simple effects analysis revealed that the interaction was driven by the significantly higher HR in the stress group during treatment (*HR*_*during*_) compared with that in the control group (*p* < .001) (see Table 1).

**Table 1:** Mean values (±*SD*) for the heart rate (HR), negative affect (NA), and positive affect (PA) measured before, during, and after treatments in the stress group and the control group.

		Test period				
	Group	Pre	During	Post1	Post2	Post3
HR (bmp)	Stress	69.5 (±7.8)	88.3 (±12.5)	73.1 (±9.8)	73.0 (±9.0)	72.3 (±8.2)
	Control	69.9 (±10.2)	75.4 (±9.7)	71.5 (±9.3)	71.4 (±9.5)	70.3 (±9.2)
NA	Stress	14.8 (±4.2)	–	17.8 (±6.2)	15.1 (±4.7)	14.2 (±4.1)
	Control	15.8 (±4.0)	–	14.8 (±3.8)	13.1 (±3.2)	13.6 (±3.2)
PA	Stress	30.8 (±6.4)	–	28.3 (±6.9)	27.6 (±6.8)	27.1 (±7.4)
	Control	29.7 (±5.9)	–	29.3 (±6.9)	27.6 (±7.0)	25.6 (±7.3)

For negative affective state, the mixed two-way ANOVA revealed a significant main effect of Test period, *F*(3, 165) = 10.608, *p* < .001, partial *η*^2^ = 0.162, and a significant interaction of Test period * Group, *F*(3, 165) = 6.750, *p* < .001, partial *η*^2^ = 0.109. Simple effects analysis revealed that the interaction was driven by the significantly higher negative affect measured immediately after treatment (*NA*_*post*1_) in the stress group compared with that in the control group (*p* < .05) (see Table 1).

For positive affective state, the mixed two-way ANOVA revealed a significant main effect of Time, *F*(3, 165) = 20.872, *p* < .001, partial *η*^2^ = 0.275, but no significant main effect of Group or significant interaction of Time * Group (*ps* > .05). Post hoc comparisons found that the PA score of the first measure was higher than that of the second measure, and PA scores of the second and third measures were higher than that of the fourth measure (*ps* < .05) (see Table 1).

### Descriptive statistics of temporal perception performance

**Figure 3:**
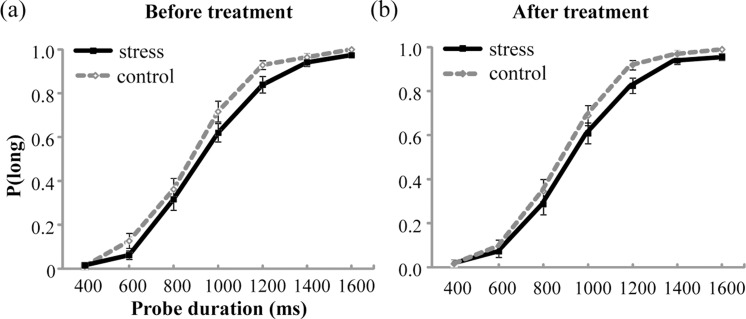
Proportion of long responses, *P*(long), was plotted against probe durations for the stress group and the control group before (A) and after (B) the treatment. Error bars are SEM.

The mean values of *P*(long) were plotted against probe durations for the stress group and the control group before and after the treatment, as shown in Fig. 3. The analysis of *P*(long) found a significant main effect of Probe duration, indicating that *P*(long) increased with the increase in probe duration, *F*(6, 330) = 668.703, *p* < .001, partial *η*^2^ = 0.924. However, no significant main effect of Group or Test period, or any significant interaction was detected (*ps* > .05).

The mean values of BP and WR are shown in Table 2. The analysis for both BP and WR did not show any significant main effect of Test period or Group, or significant interaction effect between Test period and Group (*ps* > .05).

**Table 2:** Mean (*M*) and standard deviation (*SD*) for the temporal perception performance before and after treatments in the stress group and the control group.

	Stress group				Control group			
	Before		After		Before		After	
	*M*	*SD*	*M*	*SD*	*M*	*SD*	*M*	*SD*
Bisection point (BP) (ms)	949	158	962	180	907	144	903	126
Weber ratio (WR)	0.137	0.058	0.139	0.062	0.132	0.044	0.132	0.060

### Relationship between stress responses and temporal perception performance changes

**Figure 4:**
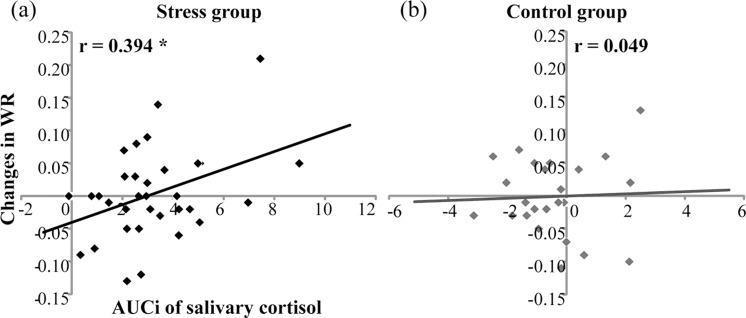
Correlation of salivary cortisol responses (AUCi) and changes in Weber ratio (WR) before and after the treatment in (A) the stress group (*n* = 33) and (B) the control group (*n* = 24). * denotes *p* < 0.05.

For temporal sensitivity, Pearson correlation found that in the stress group, AUC_i_s of salivary cortisol were positively correlated with changes in WRs (*r* = 0.394, *n* = 33, *p* < .05) (Fig. 4A). However, in the control group, AUC_i_s of salivary cortisol were not correlated with WR increases (*r* = .049, *n* = 24, *p* = .822) (Fig. 4B). For subjective duration, AUC_i_s of salivary cortisol were not correlated with changes in BPs for the stress group (*r* = .155, *n* = 33, *P* = .388) or for the control group (*r* = .307, *n* = 24, *p* = .144).

## Discussion

The present study was designed to investigate the relationship between individuals’ stress responses and time perception. The modified TSST task here elicited significant physiological responses and affective changes in the stress group compared with the control group, including cortisol increases, HR increases, and NA increases, suggesting the TSST task was effective in stress induction. The results did not find a general effect of acute psychosocial stress on both WR and BP, representing temporal sensitivity and subjective duration, respectively. However, cortisol responses to stress were found to be positively associated with changes in WR. This correlation was not observed for the control group. Also, the correlation between cortisol levels and changes in BP was not observed for both the groups.

The results found a positive correlation between cortisol responses to stress and increases in WR after stress, and this association was not observed for the control group (i.e., the association between changes in cortisol and WR after the nonstress control task). Note that larger WR indicates lower temporal sensitivity. This correlation suggested that individuals with stronger cortisol responses to stress had more severe deficits in temporal sensitivity. This finding is in line with previous studies that found correlations between cortisol stress responses and cognitive components of time perception (Church, 1984; Zakay & Block, 1997; Grondin, 2010; Kopec & Brody, 2010). For instance, stress-induced cortisol elevations have been found to be associated with poorer working memory performance (Qin et al., 2009; Schoofs, Wolf & Smeets, 2009), larger long-term memory impairments (Buchanan & Tranel, 2008; Smeets, 2011), and greater decision-making alterations (Starcke et al., 2011). Both the memory and decisional components play critical roles in time perception (Grondin & Rammsayer, 2003). The present results provide direct evidence for the negative association between stress response and temporal sensitivity. However, the association between changes in cortisol and temporal sensitivity was not observed in the control group, although the AUCi of the salivary cortisol levels within the control group also showed individual variability. These results suggest that cortisol responses to stress but not basal cortisol variations were correlated with temporal sensitivity.

The mechanism behind the association between the cortisol response to acute stress and temporal sensitivity is poorly understood. On the one hand, the high stress or stress hormone level may have an effect on temporal sensitivity. In line with this inference, previous studies have found that cognitive components of time perception, such as attention (Braunstein-Bercovitz, 2003; Paczynski, Burton & Jha, 2015), working memory (Duncko et al., 2009; Qin et al., 2009; Schoofs, Wolf & Smeets, 2009) and long-term memory (Buchanan & Tranel, 2008; Wolf, 2008; Smeets, 2011; Schwabe & Wolf, 2013), and decision-making (Van den Bos, Harteveld & Stoop, 2009), were affected by acute stress. Neuroimaging studies have also suggested that high cortisol release in response to stress has influenced the functions of brain regions supporting time perception, including the prefrontal cortex and/or hippocampus (Pruessner et al., 2008; Qin et al., 2009), and may have thus caused temporal sensitivity decreases.

On the other hand, this correlation may suggest common mechanisms mediating hypothalamic–pituitary–adrenocortical (HPA) induction to stress and supporting temporal sensitivity at the same time. The prefrontal cortex and hippocampus, where corticosteroid receptors are abundantly expressed, not only support cognitive functions related to time perception (Coull, Cheng & Meck, 2011; Gu, Van Rijn & Meck, 2015), but also play important roles in the glucocorticoids negative feedback regulation (McEwen, 2004; Ulrich-Lai & Herman, 2009). It might be the case that individuals who did worse in recruiting the prefrontal cortex and/or hippocampus for effective negative feedback regulation of the HPA axis to attenuate stress-induced increases in cortisol did worse in recruiting these areas for efficient temporal information processing to maintain sensitive discrimination of different durations at the same time.

In contrast to temporal sensitivity, changes in subjective duration as indexed by BP were not found to be related with stress responses in cortisol, which suggested a dissociation in the relationship between stress and the two elementary aspects of time perception. According to the assumption in the framework of cognitive models, the subjective duration may depend mainly on the clock stage while the temporal sensitivity may be related more closely with the memory and decision stage (Grondin & Rammsayer, 2003). The findings of the current study may indicate the dissociated effects of stress on the different processing stages of time perception. Furthermore, previous studies have suggested that these two aspects of time perception have different neurochemical mechanisms and manipulators (Santi, Weise & Kuiper, 1995; Crystal, Maxwell & Hohmann, 2003; Grondin & Rammsayer, 2003; Cheng & Meck, 2007; Rammsayer, 2010; Yin & Meck, 2014). For example, pigeons and rats showed specific declines in temporal sensitivity, but not in subjective duration, on received injections of compounds that were known to affect memory and attention (e.g., cannabinoid and amphetamine) (Crystal, Maxwell & Hohmann, 2003; Santi, Weise & Kuiper, 1995). The findings here further suggested that temporal sensitivity, but not subjective duration, is related to the HPA axis stress response in humans.

When comparing differences between the stress group and the control group, the results did not find a general effect of acute psychosocial stress on both temporal sensitivity and subjective duration. Previous studies focusing on comparing differences between the stress condition and the control condition found mixed results regarding temporal sensitivity. The results of some studies were similar to the results of the present study, showing unchanged temporal sensitivity under stress (Droit-Volet et al., 2010), while others found increases (Tamm et al., 2014) or decreases (Watts & Sharrock, 1984) in temporal sensitivity under stress. One possible explanation for these inconsistent results may be that individuals responded differently under stressful conditions and showed different behavioral patterns in temporal sensitivity. In line with this inference, when individual differences in stress responses were analyzed, an interesting correlation, as discussed in the preceding text, was detected in the present study.

However, the result regarding subjective duration was inconsistent with previous studies, which found longer subjective durations under acute stress compared with the control condition (e.g., Watts & Sharrock, 1984; Droit-Volet et al., 2010; Tamm et al., 2014). It is to be noted that subjective durations were measured after the end of the stressor here. In contrast, the previous studies utilized ongoing stressors and participants performed the time perception tasks during stress (Watts & Sharrock, 1984; Droit-Volet et al., 2010; Tamm et al., 2014). As the results showed, the heart rate reached the highest level during the stressor and recovered very fast after the stress. Considering that the heart rate has been widely used as an indicator of arousal (e.g., Angrilli et al., 1997; Hellhammer & Schubert, 2012; Pollatos et al., 2007), the differences in arousal levels might explain why significant effects of stress on subjective duration were not detected as in previous studies.

Limitations of the present study have to be acknowledged. First, in controlling for sex differences in responses to stress (e.g., Kajantie & Phillips, 2006; for a review, see Kudielka & Kirschbaum, 2005), only male participants were included. It is important for future studies to determine whether the results obtained in healthy young male adults here can be generated for females as well. Second, the duration range used in the temporal bisection task was from 400 to 1,600 ms. As different neurocognitive mechanisms have been put forward for the perception of short and long durations (Lewis & Miall, 2003; Lewis & Miall, 2006), it is still unclear whether the same pattern of results would emerge when examining time perception of longer durations.

In conclusion, the present study found that although no significant change was observed in time perception after an acute stressor on the group-level comparison (i.e., stress vs. nonstress group), healthy male participants with stronger cortisol responses to stress showed a larger decrease in temporal sensitivity. This finding may provide insight into the understanding of the relationship between acute psychosocial stress and temporal sensitivity by emphasizing the role of individual differences in stress responses.

## Supplemental Information

10.7717/peerj.2061/supp-1 Data S1 Raw data

## Additional Information and Declarations

### Data Availability

The following information was supplied regarding data availability: The raw data has been supplied as Data S1.
